# Bullying as a Developmental Precursor to Sexual and Dating Violence Across Adolescence: Decade in Review

**DOI:** 10.1177/15248380211043811

**Published:** 2021-09-14

**Authors:** Dorothy L. Espelage, Katherine M. Ingram, Jun Sung Hong, Gabriel J. Merrin

**Affiliations:** 1 University of North Carolina at Chapel Hill, NC, USA; 2 Wayne State University, Detroit, MI, USA; 3 Syracuse University, NY, USA

**Keywords:** bullying, sexual violence, teen dating violence, bully–sexual violence pathway

## Abstract

Adolescent bullying continues to be a major focus of scholarship across the globe. This article reviews research from 2010 to 2021 with a particular focus on longitudinal studies of the *bully–sexual violence pathway* (*BSVP*), where bullying serves as a precursor for sexual violence (SV) (e.g., sexual harassment, sexual coercion, and sexual assault) and teen dating violence via individual and socio-contextual mediators. Articles reviewed consisted of a total of 505, which included 17 meta-analyses and systematic reviews. Databases used for the search were Academic Search Complete, Education Full Text (H. W. Wilson), ERIC, National Criminal Justice Reference Service Abstracts, PsycINFO, PubMed (Medline), and Social Sciences Abstracts (H. W. Wilson). In total, 107 peer-reviewed articles were included in this review. Potential mechanisms underlying the *BSVP* include social dominance orientation, exposure to sexual education, and alcohol use. Several school-based intervention approaches have evidenced marginal success in reducing rates of bullying and SV by targeting factors undergirding both behaviors. The efficacy of international prevention approaches is summarized. Gaps in the literature are identified and future research is proposed.

Adolescence is a developmental period between 10 and 19 years of age, often of growing independence for young people (Health & Human Services, 2016; World Health Organization, 2014). Adolescents tend to spend increasing amounts of time with similar-age peers who are also experiencing this critical period of identity development, growth, risk-taking, and maturation. This developmental period also brings about exposure to peer aggression in numerous forms, including bullying and sexual violence (SV) involving other students who may be peers, friends, or romantic partners. Since the 1980s, there has been an increase in research on aggression involving children and adolescents (López et al., 2008). Although conflict with peers is a typical part of social development in adolescence, harmful aggressive behaviors such as bullying and SV can reinforce psychological and physical harm for adolescents (Gruber & Fineran, 2016; Okafor et al., 2020). These phenomena have been studied in many parts of the world such as the United States (Espelage et al., 2018a; Zych et al., 2019), Canada (Humphrey & Vaillancourt, 2020; Zych et al., 2019), Europe (Hellevik & Øverlien, 2016; Viejo et al., 2020; Zych et al., 2019), and South Africa (Shamu et al., 2016; Zych et al., 2019). The scholarship has proliferated in recent years on how and under what conditions bullying is followed by SV on a continuum of aggressive behaviors as youth age (Basile et al., 2009; Zych et al., 2019). Researchers are increasingly finding that adolescents who are involved in bullying are at an elevated risk of various forms of SV, including sexual harassment, sexual assault, and teen dating violence (TDV). Given the empirical literature that has amassed over the past decade, it is an appropriate moment for review, synthesis, reflection, and discussion about integrating findings into practice and policy and highlighting future directions for research.

## Current Review of the Literature

This article reviews research published from 2010 to 2021 with a particular focus on longitudinal studies of the bully–sexual violence pathway (BSVP) where bullying serves as a precursor for SV (e.g., sexual harassment, sexual coercion, sexual assault) and TDV via individual and socio-contextual mediators. Articles reviewed consisted of a total of 505, which included 17 meta-analyses and systematic reviews. Inclusion criteria are (a) early to late adolescent sample, (b) peer-reviewed article, and (c) published between 2010 and 2021. Search terms included bullying, SV, sexual harassment, TDV, adolescent relationship aggression, dating violence, homophobic teasing, homophobic name-calling, biased-based bullying, and intimate partner violence. Reference harvesting was conducted until saturation was achieved in that we were not finding any new articles. Databases used for the search included Academic Search Complete, Education Full Text (H. W. Wilson), ERIC, National Criminal Justice Reference Service Abstracts, PsycINFO, PubMed (Medline), and Social Sciences Abstracts (H. W. Wilson). In total, 107 peer-reviewed articles were considered in this review. Also included in these advancements is the exploration of the risk and protective factors that might amplify or attenuate the associations between bullying and SV. We highlight theories that link adolescent bullying with SV and TDV, with a focus on mediators of the BSVP with a gendered and equity lens.

We provide definitions of the constructs of interest, discuss theories linking bullying to SV, and end with illustrations of the few prevention programs that target these phenomena concurrently.

## Adolescent Bullying

Bullying has been conceptualized as a specific type or pattern of one or more forms of aggression that is relational in nature. This phenomenon has been an international focus of scholarship for several decades (Hong & Espelage, 2012; Rodkin et al, 2015; Volk et al., 2014 , 2017). The term “bullying” originated in Germany in 1538 as, “a fine chap, a hired ruffian, or a blustering browbeating person—especially one who is cruel to others who are weaker” (Volk et al., 2014, pp. 327–328). However, in the empirical literature, the most familiar and widely cited definition was conceptualized and derived by Dan Olweus (1978). Olweus (1993) first proposed and defined bullying in the 1970s as a subcategory of aggression characterized by three critical components: (1) intentionality in committing the aggressive act, (2) repetition or fear of repetition, and (3) a power imbalance where perpetrators have some advantage over their victims (e.g., physical size or strength, status, competence, social influence) where victims would have difficulty defending themselves (Olweus, 1993). Some research suggests that when all three components (repetition, severity, and a perceived or observed power imbalance) are present in an incident where aggression is involved, there is an amplification of harm inflicted upon the target (Kaufman et al., 2020; Van der Ploeg et al., 2015; Van Noorden et al., 2016; Ybarra et al., 2014). This distinction separates bullying from a general aggressive act.

Within the last decade, scholars have elaborated on the definitions of bullying in an attempt to examine adolescents’ motivations for engaging in bullying. Volk et al. (2014) proposed a similar definition to Olweus with a few augmentations, by including a proposition that harm done to the victim (including fear of future harm) is a more important and useful indicator of the presence of bullying than other components. Their definition focuses more on bullying being a goal-directed behavior (i.e., the agent gains something by perpetrating harm against another person), as bullying largely involves proactive aggression rather than reactive aggression (Dodge & Coie, 1987). Often the goals served by bullying are related to social dominance, which can include competing for social influence in a specific context (e.g., school, team, workplace, community group), or another seemingly limited social resource like the attention of a peer or dating partner (Hawley, 1999; Hawley & Bower, 2018). Strong support for this social dominance component of bullying is reflected in the extant literature (Caravita & Cillessen, 2012; Garandeau et al., 2014). In a 2011 study of 9- to 12-year-old children, Oltof et al. (2011) found that children engaged in bullying employed both coercive and prosocial strategies and the most actively-involved bullies (i.e., ringleader bullies) expressed the highest social dominance orientation, which was measured by a validated psychometric scale. Taken together, it appears that there is an agreement that harm to a victim should be the primary concern in any bullying situations. Also, intentionality or goal-directedness distinguishes bullying from an act of aggression that may be unintentional or serve no purpose for the agent, such as a reactive haughty verbal response to a peer because of a bad mood. Several studies show a fair amount of stability in class membership over time such that students who primarily assume only one role (either bullying others or are victimized) are likely to remain in these roles across time (Zych et al., 2020). However, students who simultaneously bully others and are victimized experience a higher level of role fluctuation over time (Zych et al., 2020).

Rates vary by nation, but bullying appears to be a cross-cultural phenomenon with worldwide empirical evidence (Bradshaw et al., 2017; Salmivalli, 2018; Smith et al., 2016). According to the National Center for Education Statistics, 24% of students in public schools in the United States reported bullying among students at least once a week in 2017–2018 (Wang et al., 2020). Bullying perpetration estimates vary but, in general, reveal that 17.3%–31.8% of middle and high school students in the United States report face-to-face bullying perpetration in the past few months (Kowalski & Limber, 2013; Kowalski et al., 2012). Also, according to the United Nations Educational, Scientific and Cultural Organization (UNESCO, 2017), children and adolescents affected by school bullying vary between countries and research findings, ranging from less than 10% to over 65%. UNESCO (2018) also documented that the highest rate of physical bullying was reported in the Pacific and sub-Saharan Africa, and the highest rate of sexual bullying was reported in Central America, the Middle East, and North Africa. In many countries, the rate of bullying has declined over time, but fewer countries have reported a decrease in physical violence (UNESCO, 2018).

While context-specific dynamics may vary, students who most often experience victimization across the world are those who are perceived as different, negatively different, or weak (UNESCO, 2018). For example, racism, misogyny, ableism, and heteronormativity in Western cultures place racial and ethnic minority students, nonbinary and transgender students, and students with disabilities at risk for bullying victimization by others (Juvonen & Graham, 2014). Additionally, across the globe, students whose families have less wealth or are considered low socioeconomic status face a high risk of being victimized (Tippett & Wolke, 2014). Regarding qualities common to perpetrators, evidence from studies in several countries suggests boys are more likely to engage in physical or cyberbullying than girls, but girls are more likely to engage in traditional relational aggression or social bullying (Hu et al., 2021; Wang et al., 2020). A recent study conducted by Hu et al. (2021), which included a sample of adolescents from China, found that while both males and females reported engaging in bullying, males engaged in more traditional and cyberbullying, while females were more involved in traditional and relational bullying.

## Sexual and Dating Violence Among Adolescents

### Sv and Cyber SV

SV refers to any sexual act, verbal or physical, that one (or more) individual(s) enacts against another who does not or cannot freely and enthusiastically consent (Basile et al., 2016; World Health Organization, 2011). SV is perpetrated by and against adolescents all over the world (Vanwesenbeeck, 2008). Examples of SV include lewd or harassing remarks about one’s body or sexuality, sharing nude or intimate photos of someone without their consent, sexual threats, and unwanted, forced, or coerced sexual contact (Espelage et al., 2016a). Also, homophobic teasing or taunting is a form of SV, as it is intended to call attention to a queer or a perceived queer sexual orientation in a pejorative way, that asserts heteronormativity and thus dominance for those nearby to witness (Rivers, 2011; Russell & Horn, 2017). Data from the National Intimate Partner and SV Survey indicate that over half of all sexual assault victims are acquainted or friends with the perpetrator (Smith et al., 2018). Research has conceptualized SV as another form of bullying behavior; however, although similar to bullying SV is not merely a form of bullying. It is another type of dominance-based interpersonal violence that is experienced by adolescents in many parts of the world. Scholars have recognized that SV is another global public health concern (Borumandnia et al., 2020).

SV can be perpetrated both face-to-face and via cyberspace. Over the past decade, SV through cyberspace, or cyber-SV, has also gained research attention globally (Arafa et al., 2018; Pereira et al., 2016). Based on a systematic review of the extant literature, Fernet et al. (2019) synthesized defining aspects of cyber-SV behaviors. First, cyber-SV can be direct (i.e., involving messages sent to or directly targeting an individual) or indirect, in which harmful contents are posted for viewing by a broader viewing audience (i.e., followers), thereby damaging the victim’s reputation or relationships. Examples of direct forms of cyber-SV include repeated unwanted messages, unwanted pornographic messages, and harassing comments (Fernet et al., 2019). Examples of indirect cyber-SV include nonconsensual posting of private photos or sharing of intimate anecdotes on public or semi-public forums (Fernet et al., 2019). Additionally, the capabilities of tools that are common to many devices such as location services, anonymity, and immediate access to a wide audience allow for other behaviors such as cyberstalking, violence from unknown or untraceable perpetrators, or threats leveraging the power of sharing to the greater audience.

The prevalence of SV varies widely across nations. A review of research also found that in 27 European Union countries, the lifetime prevalence rate of SV victimization involving females (excluding child sexual abuse) ranged from 9% to 83%, and the rate of SV victimization of males ranged from 2% to 66% (Krahe et al., 2014). Ybarra and Mitchell’s (2013) findings indicated that in the United States, nearly 1 in 10 adolescents nationwide have reported some type of SV perpetration. A study by Decker et al. (2014), which comprised a sample of adolescents (15–19 years of age) in five countries (United States, India, Nigeria, South Africa, and China), reported that the prevalence of SV victimization ranged from 10.2% in China to 36.6% in South Africa. The study also showed that the lifetime non-partner SV victimization ranged from 1.2% in China to 12.6% in South Africa. Variations in the rates of SV are likely due to measurement differences and error and different cultural definitions of acceptable violence. According to Kalra and Bhugra (2013), SV tends to occur more frequently in countries that foster beliefs in male superiority and dominance.

In addition to sex assigned at birth differences, a limited number of studies have found racial and ethnic differences in SV. Racism and White supremacy place youth of non-White races and ethnicities at significantly high rates of victimization. A U.S. Centers for Disease Control and Prevention survey documented that overall, about 7% of all females and 1.3% of males experience rape before age 18 (Smith et al., 2018; the report does not include data on gender nonbinary youth). However, about half of multiracial females, 46% of Native American females, and between 30% and 40% of Black and White females (21.5% overall) and about one-third of Native American males, and about 20% of Black and Hispanic males experience a form of SV that involves physical contact in their lifetimes (Smith et al., 2018). Gender nonbinary individuals were not included. For males, according to the findings from one recent literature review of the empirical research, European migrants, applicants for international protection, and refugees (MARs) are especially vulnerable to SV (Schrijver et al., 2018). The review also suggests that SV is highly frequent among MARs in Europe, despite a serious dearth of research on the experiences of SV of the MARs population and methodological limitations in the existing studies (Schrijver et al., 2018).

Differences in SV based on sexual orientation and gender identity have also been documented (Pathela & Schillinger, 2010). In a nationally representative survey of lesbian, gay, bisexual, transgender, and (sexually) questioning (LGBTQ) youth in the United States, 82% reported being the victim of verbal harassment in the past year, and nearly 40% reported having this experience “often or frequently” (Kosciw et al., 2018). The same survey found that 37% of the sample reported physical sexual harassment in the past year, and 12.4% reported physical assault based on their sexual orientation. Nearly 40% of bisexual females in another sample were victims of sexual assault (Ford & Soto-Marquez, 2016). Also, according to Kosciw et al. (2018), about half of LGBTQ students in the United States report being sexually harassed online in the past year, with about 14% reporting that the harassment was frequent.

### TDV

Romantic relationships become increasingly important to adolescents’ social lives and can bring about positive experiences of joy and support in addition to harmful experiences such as dating violence (Hutchison, 2017). TDV can include the types of SV mentioned above, in addition to any other patterns of physical, verbal, or psychological violence enacted within the context of the intimate relationship (Exner-Cortens et al., 2016). Like SV, identity and social context are critical data points in understanding how SV and TDV affect youth and communities.

The prevalence and trend of TDV vary by study findings. Wincentak et al.’s (2017) meta-analytic study of the literature on the prevalence of TDV in several countries found that about 20% of teens experience violence in a romantic relationship and about 9% experience sexual forms of TDV. One study conducted in Canada also found that although physical dating violence victimization of adolescents declined, the rates of physical dating violence victimization over the 10 years varied by sex. More specifically, compared with girls, boys showed higher rates of physical dating violence victimization in 2003, 2008, and 2013 (Shaffer et al., 2021). This finding is contrary to past studies, which found that girls and women are at a higher risk of physical dating violence victimization than boys and men. Nevertheless, the authors concluded that physical TDV remains a significant problem among Canadian adolescents (Schaffer et al., 2021 ), as it is for adolescents in other countries.

Reports suggest that about 12% of U.S. middle and high school students perpetrate cyber TDV (Zweig et al., 2013). A report from the United Nations (2015) also showed that 73% of women and girls who rely on cyber technology reported being victimized by a romantic partner online. A recent Canadian report also showed that 17% of people, age 15–29, reported experiencing cyberstalking (Hango, 2016). A review of the literature on TDV also suggests that the prevalence rate of TDV victimization is comparable across Europe and North America (Leen et al., 2013).

While these figures are alarming and justify tremendous concern, they are also likely underestimated for several reasons. Measurement of dating violence behaviors can be difficult because when conflict occurs among dating partners, an individual may act reactively violent when they would not otherwise. Similar to other forms of interpersonal violence, victims and perpetrators are not always aware that their actions merit reporting on surveys (Truman & Rand, 2010). Additionally, barriers to disclosing TDV victimization and perpetration including a lack of awareness, fear of negative social consequences (such as making the situation worse), fears of not being believed, and doubts that an intervention would occur have been well-documented in the research literature (Bundock et al., 2020; Kosciw et al., 2018).

### SV as a Product and Maintenance Tool of Power Imbalance

SV, like bullying, has been conceptualized as both a product and a maintenance tool of power imbalance. There is extensive evidence that SV is often used as a tool of domination or colonization globally (Guterres, 2017). Smith (2015) highlights an important example in the history of European settlers using SV to dominate and subjugate indigenous peoples in the present-day United States. Scholars have also long identified gender power imbalances that give rise to SV, although an intersectional lens of how multiple systems of oppression together place individuals at differential risk is critical for a full understanding of this phenomenon (Crenshaw, 1989; Pascoe & Hollander, 2016). For example, most of the research on power imbalances that foster SV has focused on White males as perpetrators and White females as victims (Ford & Soto-Marquez, 2016). In many Western or Euro-centric cultures, race as a construct and racism are present in any social phenomenon, including practices of sexual and interpersonal violence (see Davis, 1981). While White females are often victims of misogyny and gendered violence, the intersection of multiple minoritized identities must be considered. For example, Black transgender females are not only face misogyny but also racism and transphobia, which leads to higher rates of harm associated with sexual and interpersonal violence (Stotzer, 2009; Zounlome et al., 2019). Women with multiple minoritized identities face interlocking systems of oppression that together work to dehumanize and harm them, with little mainstream attention, empathy, or care that is often afforded to cisgender White females. This is just one example of many that illustrate how oppressive systems work together to place individuals at differential risk of SV victimization and perpetration based on their learning histories and their intersecting identities within their cultures.

Further, societies around the world uphold norms that normalize and justify SV such that it can continue to reflect and uphold societal and interpersonal power imbalances known as “rape culture”. These norms include a lack of education on consent, coercive social scripts, victim-blaming, and the centering of heterosexual sex and male pleasure (Armstrong et al., 2018; Chitsamatanga & Rembe, 2020). First, despite many laws requiring consent, there is a widespread lack of internalized understanding regarding what “freely and enthusiastically given consent” means (Hirsch et al., 2019). Relatedly, conversations between sexual partners about consent are also not culturally normalized. Culturally learned sexual social scripts create an understanding of heterosexual sexual intercourse where women are taught to coyly resist sex while males are often taught that pursuing a sexual partner aggressively and persistently is desirable or normal (Armstrong et al., 2018). Taken together, these heterosexist, white supremacist, patriarchal, norms create a culture of sex that is difficult to separate from SV given the overlap. Thus, many adolescents, victims and perpetrators alike, often do not realize they have been involved in an SV incident as they do not have the psychoeducation or language to make sense of their experiences.

## Pathways From Adolescent Bullying to SV and TDV

As children enter puberty, they begin to develop a sexual identity and engage sexually and romantically with others. It is unsurprising that existing patterns of interpersonal aggression, including bullying, enter this new life domain and become SV and TDV. The few studies conducted on the associations between bullying and SV and TDV before 2010 suggested these phenomena co-occur in the United States (Pellegrini, 2001), Canada (Pepler et al., 2002), and Brazil (DeSouza, & Ribeiro, 2005) and some signaled that these behaviors have shared, and also unique, risk and protective factors (Basile et al., 2009). Basile et al.’s (2009) review laid the groundwork for the proposed *BSVP Theory*. According to the *BSVP* Theory, bullying and SV perpetration are longitudinally associated with one another across early to late adolescence (Espelage et al., 2012; Espelage et al., 2015a; Espelage et al., 2018a). Studies have provided support for this theory in which bullying perpetration is associated with greater homophobic name-calling perpetration and victimization during middle school, which is consistent with other theoretical and empirical research (Basile et al., 2009; Poteat & Espelage, 2005 , 2007 ; Poteat et al., 2012). However, youth who engage in high levels of bullying during early middle school and homophobic name-calling in later middle school face greater odds of later SV perpetration in middle and high school (Espelage et al., 2015a; Espelage et al., 2018a).

Foshee et al. (2016) extended this work to include bullying perpetration as a predictor of subsequent TDV, which consisted of sexual, verbal, psychological, and physical violence in the context of a romantic partnership. The study found that approximately 70% of the adolescents reported engaging in at least one of the three forms of aggression. Also, low maternal monitoring, depressed affect, and anger reactivity were significant risk factors for these three forms of aggression. Some have found that these behaviors co-occur. Miller et al.’s (2013) longitudinal study (2010–2011 and 2011–2012 school years) used a latent transition analysis, a person-centered analysis that captures and examines patterns among indicators that exist in the data. They found latent classes of bullying perpetration and victimization TDV, and SV, and examined the stability (or instability) in classes across time. For about half of this middle school sample, bullying and SV perpetration co-occurred, and boys were overrepresented in these classes compared to girls (Miller et al., 2013). Only one small class (about 12% of the sample at each wave) was characterized by TDV, which may speak to the low incidence of “dating” in middle school. However, this class was also the “multi-problem” class, where members typically reported perpetration and victimization of all three behaviors. These findings suggest that individuals are often not static in their roles as victims or perpetrators, and these findings also perhaps capture the cycle by which victimized individuals learn to enact violence to resolve conflict, attain a goal, or as a means of survival (Doty et al., 2017; Leemis et al., 2019; Williams et al., 2013). Further, being a constant aggressor-victim in the bullying literature is not usually linked with the adaptive pursuit of dominance and power (Volk et al., 2012), thereby calling for a closer examination of these dynamics within the *BSVP* Theory.

Additionally, Cutbush et al. (2016) using longitudinal structural equation modeling found that among boys, early middle school bullying, but not SV perpetration, predicted later TDV. For girls, those who perpetrated SV earlier in middle school (but not bullying) were more likely to engage in TDV later on. However, Humphrey and Vaillancourt (2020) found no sex differences present in a cascade model where stability in bullying perpetration across early adolescents was associated with homophobic taunting, sexual harassment, and dating violence during late adolescence. Although inconsistent, these findings suggest that developmental-behavioral trends may manifest differently for boys and girls, which could be attributed to cultural definitions of gender roles.

### The Roles of Dominance Orientation, Sexuality Education, and Alcohol Use in the BSVP

Although these studies capture a descriptive pathway, a preponderance of evidence suggests that patterns of a dominance-oriented interpersonal style are one potential mechanism that undergirds this developmental transformation from bullying to SV and TDV. Some youth have learned to practice domination, physically, psychologically, or relationally, as a means of goal attainment (Rigby, 2012). Youth engage in perpetrating bullying, SV, and TDV to gain social status or secure attention or inclusion, perhaps acting out of a basic need to belong or as a survival tactic (Vanden Abeele et al., 2014; Volk et al., 2014). According to Vanden Abeele et al.’s (2014) study, which comprised a sample of Flemish adolescents, teens who were popular with the opposite sex and with a greater need for popularity were more likely to report sexting behavior. The goals associated with SV among adolescents are perhaps more complex; while peer status and attention from others are certainly still motivations, the sexual pursuit of the target of the harassment is also often a primary goal (Kaltiala-Heino et al., 2018; Kelly et al., 2012). In both cases, using violence to harm another individual’s physical being, reputation, or sense of self, thereby making them vulnerable to acquiesce is a strategy that is often reinforced by reward with few repercussions (Faris & Felmlee, 2014).

During adolescence, existing dominance-orientation interacts with sexual development (Sidanius & Pratto, 2012). Inextricable from social-sexual learning and identity development is the dismissive attitude toward sexual harassment, internalization of rape culture norms, and social scripts, which appear to conceptually mediate the *BSVP* (Espelage & De La Rue, 2013). Existing power imbalances, those that reflect internalized systemic oppression within peer communities and those created by each community’s unique social capital structure, give rise to cementing these patterns. Individuals with high social capital, and who have long maintained this status, have become accustomed to taking what they feel entitled to (including human bodies) and have seemingly many sexual options given that with status comes desirability (Armstrong et al., 2018; Birkett & Espelage, 2015b; Faris & Felmlee, 2011). Individuals with less social power are not only vulnerable to coercion given their relationship to the aggressor but struggle to name attacks as such, given misinformation they have received about sexual experiences (Schneider & Hirsch, 2018). Only 20% of U.S. students received sexual education that included the sexual experiences of queer individuals (Kosciw et al., 2018). As with many forms of violence, victims are often aware that officials will not believe or honor the stories they disclose, and meaningful change is not likely to occur. This fear and the norms that birthed it allow for these systems to continue.

Finally, alcohol use has also been identified as a facilitator of SV given its inhibitory properties. Many youth are using alcohol for the first time (and often illicitly) during the same period when they are also experimenting sexually (Kann et al., 2018). Thus, alcohol elevates the inclination to harass or assault others. Many scholars have identified that in environments like parties where alcohol is present, perpetration is more likely given that alcohol consumption decreases inhibitions and increases impulsivity, thereby impairing judgment (Abbey, 2011). These same effects of alcohol consumption also preclude an individual from being able to freely and enthusiastically consent. Unfortunately, these exact properties are also attractive to adolescents, as they provide social lubrication and a sense of relaxation. Given that adolescence is often an awkward time where individuals feel both socially uncomfortable but sexually motivated, it is, therefore, not surprising that alcohol use and interpersonal violence are highly correlated (Espelage et al., 2018b ; Temple & Freeman Jr., 2011). Van Ouytsel et al. (2016) found in a cross-sectional adolescent sample that individuals who used alcohol or drugs before having sex and perpetrated bullying were also more likely to perpetrate cyber dating abuse.

## Prevention, Intervention, and Policy Efforts

Several school-based intervention programs have evidenced marginal success in reducing rates of bullying and SV by targeting factors undergirding both behaviors (Espelage et al., 2015c; DeGue et al., 2020; Miller et al., 2012; Muñoz-Fernández et al., 2019; Niolon et al., 2019; Taylor et al., 2013; Vivolo-Kantor et al., 2021). It appears that the most effective programs have a few commonalities: involvement or leadership among community members, sufficient dosage, psychoeducation, targeting interpersonal skills, and engaging bystanders and witnesses to intervene when they see such behaviors (e.g., Connolly et al., 2015). Also, programs that target multiple levels of adolescents’ social ecology, such as individual, relationship, and community levels, have been found to be effective in reducing bullying and TDV (e.g., Vivolo-Kantor et al., 2021). First, prevention is only likely to be effective if it is culturally accordant and trusted by community members at all levels of the social ecology (Catalino et al, 2012). For example, in addition to a classroom curriculum focused on communication and conflict management for middle school students, *Dating Matters* (DeGue et al., 2020; Niolon et al., 2019) provided training to middle school parents, educators, and partnered with local public health departments to engage on a policy level in the United States. They focused on the promotion of healthy relationship practices to reduce interpersonal violence and found that schools that implemented *Safe Dates* reported about 10% less bullying and sexual harassment compared to schools that implemented another standard-of-care program in the most recent trial (DeGue et al., 2020; Niolon et al., 2019). Similarly, *Shifting Boundaries*, a six-session curriculum, which focuses on the laws and consequences for TDV and sexual harassment perpetration, also saw statistically significant reductions in SV and TDV among a middle school sample after 6 months post-intervention (Taylor et al., 2013). However, they did not involve parents or families, which may be an especially important consideration for cultures that are more family-focused or collectivist. Miller et al. (2012, 2013) found support for a coach-delivered healthy relationship development program, *Coaching Boys into Men*, where athletic coaches are trained to embed messaging related to signs of dating abuse, bystander intervention, healthy communication, and gender-equity in conversational, weekly, doses.

By contrast, there are few examples of bystander-focused programs, but *dat-e adolescence*, a researcher-implemented bystander-focused program in Spain, yielded moderate effect sizes in preventing SV, TDV, and bullying (Muñoz-Fernández et al., 2019). However, a recent meta-analysis found heterogeneity in effectiveness of bystander intervention-focused programming (Kettrey & Marx, 2020). For example, Bush et al. (2019) evaluated the *Green Dot*, a bystander-targeted program that involves program participants as possible witnesses to violence. Participants are provided with skills to lower the risk for violence by (a) recognizing potentially violent situations, (b) intervening both safely and effectively to reduce the risk of violence, and (c) speaking out against attitudes that are supportive of violence. They found that Green Dot was effective in increasing bystanding behavior and reducing attitude and behavior that are supportive of violence, and these mediators were also found to be associated with reduced SV perpetration. They observed changes in a desirable direction regarding bystander intentions. *Bringing in the Bystander* (Edwards et al., 2019) did not show effectiveness on the same metrics but did not use public opinion leader identification and took a different approach to the same type of intervention. Plausibly, bystander intervention training facilitates prevention by shifting norms and creating group contingencies that de-incentivize violent behaviors. However, these programs are not without their critiques. Many programs are grounded in classical bystander theory and have been criticized for not considering the last two decades of social psychological research on social identity and shared categorization which shows that individuals are more likely to intervene when bystanders see signs of common group identity with the victim or target of the aggression (Levine et al., 2020). These scholars call on violence researchers to apply social identity theory in bystander interventions by taking into the different identities that are at play in bystander relationships to the victims, perpetrators, and other bystanders.

The success, even despite several limitations, of these programs supports SV prevention through an interconnected risk and protective factors model, where one form of violence (SV, TDV) may decrease as a result of preventing another (bullying). Further, the focus on behavior and skill-building addresses a call put forth by De La Rue et al. (2017) to move beyond attitudinal change and focus on altering behavior. De la Rue et al.’s (2017) meta-analytic review of the literature found that school-based programs influence knowledge and attitudes concerning dating violence; however, the results suggest that programs have a moderate effect on behaviors. Although the results of this review are encouraging, they also highlight the need for including skill-building components and addressing bystanders’ roles. Further, the programs reviewed here were all school-based intervention. As such, future reviews should highlight community or family-based attempts to reduce bullying, SV, and TDV.

## Where Do We Go From Here?

Findings from this review have implications for future research and practice. First, there is a critical need for more national and cross-national empirical research on the definitions and conceptualization of bullying to establish consensus on meaningful language delineations between types of aggression. One way this can happen is with more varied and comprehensive measurements of how and under what circumstances bullying, sexual, and dating violence might occur and lead to adverse psychosocial and health outcomes of adolescents. The U.S.-based Youth Risk Behavior Surveillance System (YRBS) has been helpful for broadly understanding the approximate prevalence of these actions as a surveillance tool (Centers for Disease Control and Prevention, n.d.), but assesses each form of youth violence with one or two items. Diverse measurement techniques are needed to supplement the data that can be captured with nationally representative surveys like the YRBS. For example, experience sampling methods allow for data collection in real time as incidents occur (Larson & Csikszentmihalyi, 2014). Although the method may focus on a shorter time frame, it may offer more specificity of a violent incident’s context, frequency, and immediate harm to the victim that the YRBS cannot. Further, qualitative or other research methods allow for the grounded building of knowledge regarding new forms of youth violence (e.g., TikTok in bullying) as they emerge. For instance, one study, which reviewed children’s conversations on Twitter, revealed that cyberbullying increased in the aftermath of the COVID-19 pandemic (Babvey et al., 2020). While self-reported measurement is often necessary and useful in understanding these experiences, it also brings social desirability and other biases to the data. Using behavioral tasks, observational measures, and social network analyses to supplement the many self-reported measures in this literature can add another lens to the holistic understanding of dominance-based youth violence to inform prevention efforts.

Although interventions that address bullying, SV, and TDV are increasing in number, the dire need for such multi-component, theoretically grounded, rigorously evaluated interventions remains (Espelage, 2016a, 2016b; Earnshaw et al., 2018). Assessments in research, practice, and policy of bullying and SV also need to consider societal systems of oppression (e.g., white supremacy, misogyny, homophobia, transphobia, xenophobia, ableism) as a lens through which they view youth aggression (Garnett et al., 2014; Haines-Saah et al., 2016). Biases and a lack of awareness hinder individuals in power (i.e., educators, researchers) from adequately identifying and addressing victimization experiences of adolescents with marginalized and oppressed identities (Nappa et al., 2017) need to be addressed in prevention work. Researchers and practitioners alike need to acknowledge the role of culture in producing differential victimization and perpetration experiences among youth by using measures that better allow for capturing non-White experiences. Thus, to move forward with studying youth aggression, it is imperative to give voice to those most affected and least heard, to understand how to conceptualize aggression and where to focus.

For instance, “roasting” is a type of teasing that is used among African American adolescent peer groups (Rivers & Espelage, 2013). Roasting is an aggressive oral test of linguistic creativity and verbal expressiveness that can be defined as talking about someone in a group of three or more people through clever insults (Smitherman, 1977). Played for fun or to be mean, this Black oral tradition dates back to the slavery era in the United States (Smitherman, 1977). The insults are not meant to be taken seriously; however, as the content of roasting includes personalized attacks, the lines blur between what is for fun and what is not (Smitherman, 1977, 2000). Additionally, although youth who identify as biracial or multiracial tend to be singled out by their monoracial peers due to perceived differences (see, e.g., Hong et al., 2021), there has been a serious dearth of research and intervention programs that are culturally relevant to these adolescents.

Prevention programming deployed in schools and other youth-serving settings must disrupt patterns where dominance behaviors (e.g., bullying, SV) are rewarded, and instead foster cultures of collective care, empathy, and psychoeducation regarding the ways that oppressive systems shape interpersonal interactions. As studies show, bullies and aggressive youth place importance on dominance, and they seek to acquire it; through a display of dominance, bullies are perceived as popular and powerful by their classmates even if they are not personally liked by many classmates (Menesini & Salmivalli, 2017; Olthof et al., 2011; Reijntjes et al., 2016). Thus, educators must first become aware of such dominance behaviors and disincentivize them in their own contexts, as mentioned in the previous two paragraphs. Part of this process includes teaching schoolteachers and other adults that this form of education is part of their role as members of the school community.

## Conclusion

In the last decade, scholars have identified a developmental cascade of dominance-oriented aggressive behaviors. U.S.-based researchers have called this phenomenon the *BSVP*, though importantly, it appears to have cross-cultural empirical support. International researchers have extended this pathway to include TDV, and increasingly researchers are attempting to identify the potential mechanisms underlying bullying, SV, and TDV. Interrupting this developmental continuum of aggressive behavior is an important venture. Given the importance of the peer ecology and how these behaviors are aimed to establish dominance, bystander-focused interventions vary widely in their nature and success but demonstrate some efficacy, especially in individualism-based cultures. Attention to environmental features and moderators, such as alcohol or skills training, that is conducive to violence occurring may benefit from such interventions. This review cites literature demonstrating the underlying forces of dominance-orientation and systems of misogyny. Efforts to dismantle these forces, however, show up in a culture, are much more difficult and require ongoing work for all community members. It is worthwhile, as logically follows, that radical approaches can prevent several violent behaviors (rather than only bullying or only SV). In sum, this review of the research literature provides guidance to researchers and school practitioners in disrupting the bullying-SV-TDV pathways.
